# Deuterium Isotope Effects on Acid-Base Equilibrium of Organic Compounds

**DOI:** 10.3390/molecules26247687

**Published:** 2021-12-20

**Authors:** Meiyi Liu, Jiali Gao

**Affiliations:** 1 School of Chemical Biology and Biotechnology, Peking University Shenzhen Graduate School, Shenzhen 518055, China; jiali@jialigao.org; 2 Institute of Systems and Physical Biology, Shenzhen Bay Laboratory, Shenzhen 518055, China; 3 Department of Chemistry and Supercomputer Institute, University of Minnesota, Minneapolis, MN 55455, USA

**Keywords:** deuterium isotope effects, KIE on *pK_a_*, path integral free-energy simulations, nuclear quantum effects

## Abstract

Deuterium isotope effects on acid–base equilibrium have been investigated using a combined path integral and free-energy perturbation simulation method. To understand the origin of the linear free-energy relationship of $\Delta pK_{a}=pK_{a}^{D_{2}O}-pK_{a}^{H_{2}O}$ versus $pK_{a}^{H_{2}O}$, we examined two theoretical models for computing the deuterium isotope effects. In Model 1, only the intrinsic isotope exchange effect of the acid itself in water was included by replacing the titratable protons with deuterons. Here, the dominant contribution is due to the difference in zero-point energy between the two isotopologues. In Model 2, the medium isotope effects are considered, in which the free energy change as a result of replacing H_2_O by D_2_O in solute–solvent hydrogen-bonding complexes is determined. Although the average $\Delta pK_{a}$ change from Model 1 was found to be in reasonable agreement with the experimental average result, the $pK_{a}^{H_{2}O}$ dependence of the solvent isotope effects is absent. A linear free-energy relationship is obtained by including the medium effect in Model 2, and the main factor is due to solvent isotope effects in the anion–water complexes. The present study highlights the significant roles of both the intrinsic isotope exchange effect and the medium solvent isotope effect.

## 1. Introduction

Organic and inorganic acids are stronger acids in water (H_2_O) than they are in deuterium oxide (D_2_O) [1,2] e.g., differing uniformly by about half a $pK_{a}$ unit for oxygen-containing acids [3,4]. Partly because of this difference, solvent isotope effects can have notable influences on the kinetic and equilibrium constants of chemical and enzymatic reactions [5], which, in turn, provide direct information on the mechanisms and reaction pathways [5,6,7,8]. The measurements of rates and exchange sites of proteins in heavy water or in a mixed H_2_O and D_2_O solvent have been widely used as a probe to shed light on protein dynamics and fluctuations. Moreover, recent studies by Schramm et al. further introduced complete isotopologues of enzymes—isotope substitution of proteins by replacing all naturally abundant elements with heavier isotopes (e.g., ^13^C, ^15^N, ^18^O, and, of course, ^2^H) [9,10,11]. Since the potential energy surface is not dependent on the atomic mass, the change in kinetic and equilibrium constants of these “Born–Oppenheimer enzymes” can be attributed to protein dynamics effects [12]. Nevertheless, the change in acidity of titratable amino acids, particularly those in the active site, can also alter enzyme activity. In other fields, the formation of geological deposit and the ^18^O enrichment of tree-rings are sensitive to the environmental conditions, and analyses of isotope effects can yield a historical record of the local climate changes [1]. Consequently, the study of solvent isotope effects is important, and the development of computational methods for the investigation of solvent isotope effects on acidity constants can be useful for understanding the mechanisms of enzymatic reactions [13].

In general, there is strong tendency for the $pK_{a}$ difference, $\Delta pK_{a}$, to increase with $pK_{a}^{H_{2}O}$ [14]. Different functional groups typically exhibit noticeably different trends in the magnitude and range of deuterium isotope effects. Two important factors have been proposed to contribute to the observed acidity change of organic acids, including (1) the difference in zero-point energy of the acids in water and in heavy water and (2) isotope effects on intermolecular interactions between solute and solvent molecules [3,4,15,16,17]. The former is due to the isotope exchange effect of the acid, corresponding to the replacement of a protium (considering only one titratable site) of the acid by a deuterium, whereas the latter is a medium effect as a result of the removal of the solute (acid and its conjugate base) from water into heavy water [3,4]. Both factors—in (1) the change in vibrational frequency associated with the acidic bond by isotope substitution and in (2) the difference in intermolecular interactions in water and heavy water—are dependent on the bond order of the acid and the strength of the hydrogen bonds, which qualitatively account for the dependence of $\Delta pK_{a}$ on acidity itself. However, as Schowen and Schowen pointed out, the medium effect is “as a rule vastly less important” than the exchange effect [1]. Indeed, different functional groups exhibit the somewhat characteristic magnitude of the solvent isotope effects (for example oxygen and sulfur acids), but linear dependence of $\Delta pK_{a}$ vs. $pK_{a}^{H_{2}O}$ has been reported [3,4,16,17]. Deviation from the linear free-energy relationship has been attributed to specific stereoelectronic and features such as intramolecular hydrogen bonds and charge transfer [1,3,4].

**Figure 1:**
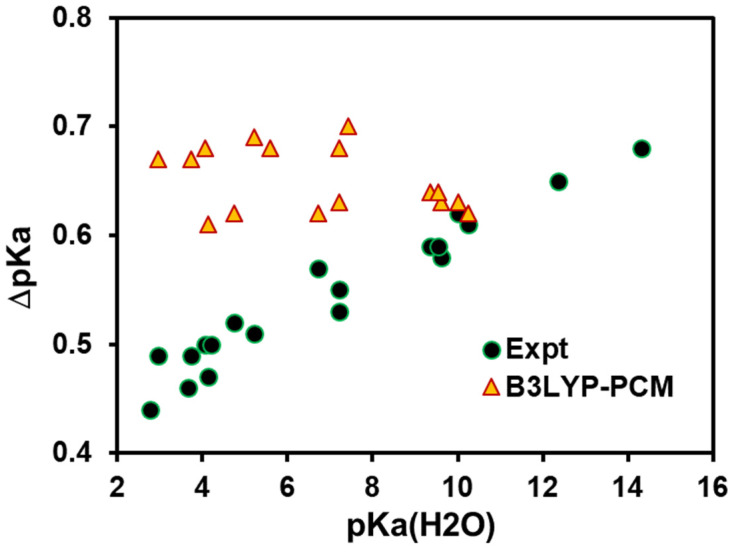
Experimental and computed deuterium isotope effects on acidity constants. The computational results determined by using density functional theory and polarizable continuum solvation model with B3LYP-PCM/6-311++G(d,p) are taken from reference [19].

Surprisingly, there have been few computational studies of solvent isotope effects on acid–base equilibria in deuterium oxide [18] except for a recent investigation by Mora-Diez et al. on a series of 11 phenol derivatives, acetic acid, and four pH indicators using density functional theory and a polarizable continuum solvation model (PCM) [19]. This comprehensive study examined several computation methods, including the treatment of the electronic structure of the acids and bases, and the representation of solvation both in water and in heavy water at different temperatures. Of the models examined, it was found that the B3LYP-IEF-PCM/6-311+G(d,p) level of theory yielded *pK_a_* values in accordance with experiments both in water and in heavy water using geometries optimized either with or without solvation effects. In particular, at ambient conditions (25 °C), a mean $\Delta pK_{a}$ value of 0.65 was obtained, which may be compared with that of 0.53 from experiments [19]. However, further examination of the aforementioned trends of isotope effects on acidity constants from experiments and computation reveals that there is a clear difference between the two sets of reported data (Figure 1). Although the average is in reasonable accordance with the experimental data, the computed $\Delta pK_{a}$ values do not exhibit a clear dependence on the $pK_{a}^{H_{2}O}$ of the acids when they are plotted together in Figure 1. We were curious about the origin of this discrepancy and thus have designed a combined path integral and free energy perturbation (PI-FEP) simulation protocol to understand the relationship between isotopic acidity shifts and the strengths of the acids, and to assess the computational procedures required to accurately determine solvent isotope effects on acidity constants [20].

A closely related study of deuterium isotope effects was the interesting observation of an increased binding affinity of histamine for its H_2_ receptor by deuteration [21]. It was suggested that hydrogen bonding interactions were strengthened upon displacement of the acidic protons by deuterons thanks to the Ubbelohde effect [21,22,23]. The heavier isotope of hydrogen tends to be more localized, having a shorter donor X-D distances relative to X-H (X = N or O), hence giving rise to weaker dipole-charge interactions and a weaker hydrogen bond than the donor atom is a proton. The authors rationalized that an N-D bond is shorter by 2.3% than the corresponding N-H as an approximation to the nuclear quantum effects and found that the relative computed hydrogen bonding energies by this constraint are consistent with the observed isotope effects.

In the following, we first highlight the computational strategy and theoretical models for determining $\Delta pK_{a}$ difference in water and in heavy water and the PI-FEP method for obtaining the ratio of the partition functions due to isotope substitutions in molecular dynamics simulations. This is followed by a description of the computational details. Then, we present and discuss the computational results along with a summary of the main findings of this study.

## 2. Theoretical Background

### 2.1. Theoretical Models for Solvent Isotope Effects on Acid–Base Equilibrium

**Scheme 1:**
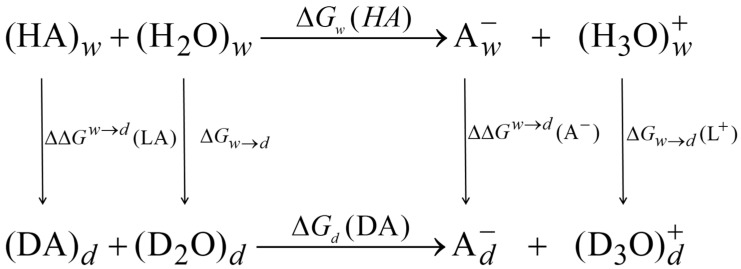
Thermodynamic cycle depicting acid–base equilibrium in water and in D_2_O.

The $pK_{a}$ difference for an acid AH in water (H_2_O) and in deuterium oxide (D_2_O), $\Delta pK_{a}=pK_{a}^{D_{2}O}-pK_{a}^{H_{2}O}$, can be determined with the aid of the following thermodynamic cycle (Scheme 1) [24].

(1) $$\begin{array}{cc} \Delta G_{d}(\mathrm{DA})-\Delta G_{w}(\mathrm{HA})= & \Delta \Delta G^{w\to d}(A^{-})-\Delta \Delta G^{w\to d}(\mathrm{LA})\\ & +\Delta G_{w\to d}(L^{+})-\Delta G_{w\to d} \end{array}$$

where L = H or D, $\Delta \Delta G^{w\to d}(\mathrm{LA})$ and $\Delta \Delta G^{w\to d}(A^{-})$ are differences in Gibbs free energy, i.e., solvent isotope effects, of the acid LA and its conjugate base $A^{-}$ in water and in heavy water, and $\Delta G_{w\to d}$ and $\Delta G_{w\to d}(L^{+})$ are, respectively, the difference in nuclear quantum effects (NQE) between water and D_2_O and between a proton and a deuteron. The latter quantities are intrinsic properties of the solvents, and they do not change when different acids are studied [18]. Thus, the difference in equilibrium constants ($K_{a}(\mathrm{DA})-K_{a}(\mathrm{HA})=\exp[-\{\Delta G_{d}(\mathrm{DA})-\Delta G_{w}(\mathrm{HA})\}/RT]$) for the ionization of the acid LA (L = H or D) in H_2_O and in D_2_O can be related to the isotope effects on the relative free energies of solvation (Equation (1)).

NQEs between H_3_O^+^ and D_3_O^+^ are difficult to be evaluated precisely using a computational method [18]. To circumvent this problem, we consider a second acid, LB (L = H and D), for which an identical equation as Equation (1) can be written [24]. By taking their difference and noting that $2.303RT\Delta pK_{a}(\mathrm{LA})=$$\Delta G_{d}(\mathrm{DA})-\Delta G_{w}(\mathrm{HA})$, we obtain

(2) $$\begin{array}{ll} \Delta pK_{a}(\mathrm{LA})-\Delta pK_{a}(\mathrm{LB})= & \frac{1}{2.303RT}\{[\Delta \Delta G^{w\to d}(\mathrm{LB})-\Delta \Delta G^{w\to d}(\mathrm{LA})]\\ & +[\Delta \Delta G^{w\to d}(A^{-})-\Delta \Delta G^{w\to d}(B^{-})]\} \end{array}.$$

Therefore, the solvent isotope effects on the acidity difference of acid LA can be related to that of acid LB by

(3) $$\Delta pK_{a}(\mathrm{LA})=\frac{1}{2.303RT}\{\Delta G(\mathrm{LB})-[\Delta \Delta G^{w\to d}(\mathrm{LA})-\Delta \Delta G^{w\to d}(A^{-})]\}$$

where ΔG(LB) is defined by combining the experimental and computational data for the acid LB in Equation (2) as follows:

(4) $$\Delta G(\mathrm{LB})=2.303RT\Delta pK_{a}(\mathrm{LB})+\{\Delta \Delta G^{w\to d}(\mathrm{LB})-\Delta \Delta G^{w\to d}(B^{-})\}.$$

Thus, ΔG(LB) can be treated as a constant using Equation (4), employing the experimental acidity shifts of acid LB, $\Delta pK_{a}(\mathrm{LB})$, and the computed free energy transfer from water into heavy water (see below). Throughout this work, we have used acetic acid ($\Delta pK_{a}=0.52$) [4,19,25] and 1 molar concentration as the reference state to determine the $pK_{a}$ differences of other acids.

It is interesting to note that we can separate each of the free energy differences in Equation (3) into two terms: one due to the exchange of a proton by a deuteron (exchange effect) at the titratable site (the acid), and one due to the difference in solute–solvent interactions (medium effect) [1]:

(5) $$\begin{array}{l} \Delta \Delta G^{w\to d}(\mathrm{LA})-\Delta \Delta G^{w\to d}(A^{-})=[G({\left\{\mathrm{DA}\right\}}_{D_{2}O})-G({\left\{\mathrm{HA}\right\}}_{H_{2}O})]\\ +[\Delta G_{\mathrm{ME}}^{D_{2}O}(\mathrm{DA})-\Delta G_{\mathrm{ME}}^{H_{2}O}(\mathrm{HA})]-[\Delta G_{\mathrm{ME}}^{D_{2}O}(A^{-})-\Delta G_{\mathrm{ME}}^{H_{2}O}(A^{-})] \end{array}$$

where the free energy of the conjugate base has dropped out since there is no isotopic exchange for the anion. The first difference on the right-hand side of Equation (5) in square brackets defines the “exchange effect” of the acid itself (in different media) as described by Schowen and Schowen [1]. The last two differences correspond to the “medium effect”, or isotope effects on solute–solvent interactions. Since the medium effect has relatively small contributions to the overall acidity shifts compared with the exchange effect, it allows us to construct two theoretical models: one that neglects the medium effect, and the second that includes the medium effect [1].

1. Model 1: Since the difference $\Delta pK_{a}(\mathrm{LA})$ is computed relative to that of $\Delta pK_{a}(\mathrm{AcOL})$, the small isotope effects in different media in each term of Equation (5) can be all lumped into the constant reference free energy term in Equation (4), ΔG(LB)≡ΔG(AcOL), as long as the computational results for AcOL are obtained in exactly the same way as that for other acids. Then, the Model-1 (M1) $\Delta pK_{a}(\mathrm{LA})$ is determined by
  (6a) $$\Delta pK_{a}^{M1}(\mathrm{LA})=\frac{1}{2.303RT}\{\Delta G(\mathrm{AcOL})-\Delta G_{\mathrm{EE}}^{w\to d}\}$$
  where ΔG(AcOL) has a combined value of −0.7895 kcal/mol in the present study (Table 1), and $\Delta G_{\mathrm{EE}}^{w\to d}$ is the free energy difference associated with the exchange effect (EE) of acid LA:
  (6b) $$\Delta G_{\mathrm{EE}}^{w\to d}=G_{H_{2}O}(\mathrm{DA})-G_{H_{2}O}(\mathrm{HA})$$
2. Model 2: The isotope effects on the med ium terms (Equation (5)) can be incorporated into $\Delta pK_{a}(\mathrm{LA})$ calculations by approximating the NQE with the inclusion of one solvent molecule that directly forms a hydrogen bond with the acid and its conjugate base explicitly. Therefore, Model 2 (M2) $\Delta pK_{a}^{M2}(\mathrm{LA})$ can be expressed as follows:
  (7a) $$\Delta pK_{a}^{M2}(\mathrm{LA})=\Delta pK_{a}^{M1}(\mathrm{LA})+\frac{1}{2.303RT}\Delta \Delta G_{\mathrm{ME}}^{w\to d}$$
  where $\Delta \Delta G_{\mathrm{ME}}^{w\to d}$ is the free energy difference due to the medium effect (ME) on acid and base interactions with the solvent:
  (7b) $$\begin{array}{cc} \Delta \Delta G_{\mathrm{ME}}^{w\to d}= & [G(D_{2}O\cdots \mathrm{DA})-G(H_{2}O\cdots \mathrm{HA})]-[G(H_{2}O\cdots \mathrm{DA})-\\ & -G(H_{2}O\cdots \mathrm{HA})]-[G(\mathrm{DOD}\cdots A^{-})-G(\mathrm{HOH}\cdots A^{-})] \end{array}$$

Before concluding this section, we note that the study by Mora-Diez et al. [19] was performed with the use of Equation (6a), i.e., Model 1 for computing the solvent isotope effects, although there are two main differences with the present work: (1) the absolute pKa values for the acids in water and in heavy water were determined by using density functional theory (DFT) calculations and the use of an experimental solvation energy for the proton, and (2) solvation effects are treated by a dielectric continuum model modified to take into account of the mass and density variations. Of course, continuum solvation models do not provide specific hydrogen-bonding interactions, unless a specific solvent molecule is included. Mora-Diez et al. found that the dominant factor contributing to $\Delta pK_{a}$ is the difference in zero-point energy of the acids, and the effects from thermal vibrational correction as well as isotope effects on the solvation of the acid and its conjugate base are negligible (i.e., $\Delta G_{sol}^{D_{2}O}(\mathrm{DA})-$$\Delta G_{sol}^{H_{2}O}(\mathrm{HA})\approx 0$ and $\Delta G_{sol}^{D_{2}O}(A^{-})-$$\Delta G_{sol}^{H_{2}O}(A^{-})\approx 0$), giving rise to the a weak dependence between $\Delta pK_{a}$ and $pK_{a}^{\mathrm{HA}}$ (Figure 1) [19]. Nevertheless, as will been seen below, the observed dependence of isotope effects on the acidity constant in fact originates from the specific interactions that are omitted in Model 1 but included in Model 2.

### 2.2. Isotope Effects from Path Integral-Free Energy Perturbation Simulations

The free energy difference between different isotopologues in Equations (6b) and (7b) defined in each pair of square brackets is related to the ratio of the quantum mechanical partition functions of two isotopic isomers, which can be directly obtained from path integral-free energy perturbation (PI-FEP) theory in molecular dynamics simulations in an explicit solvent [34]. In general, the equilibrium isotope effect (EIE), i.e., the Gibbs energy difference, between a species containing the ^2^H (D) isotope, *X*[D], and that of *X*[H] replaced by a hydrogen atom ^1^H (H), is given by

(8) $$\Delta G_{\mathrm{EIE}}=G(X[D])-G(X[H])=-RT\ln\frac{Q(X[D])}{Q(X[H])}$$

where Q(X[D]) and Q(X[H]) are the isotope-dependent quantum mechanical partition functions for *X*[L], respectively, containing D and H isotopes [20]. A key feature to achieving the necessary precision for the small free energy difference in Equation (8) is to determine the ratio Q(X[D])/Q(X[H]) through a *single simulation*, as [35] first described in [34]. This procedure relies on the use of free energy perturbation (FEP) theory [36] to treat the mass of the proton (H) as a perturbation variable to the heavier isotope (D) in path integral sampling (see below):

(9) $$\frac{Q(X[H])}{Q(X[D])}=<e^{-\beta \{V_{eff}(X[H])-V_{eff}(X[D])\}}{>}_{X[D]}$$

where $\beta =1/(k_{B}T)$ with $k_{B}$ being Boltzmann’s constant and T being the absolute temperature, $V_{eff}$ being the quantum-mechanical effective potential [37] for the isotopologue indicated in parentheses, and $<\cdots {>}_{X[D]}$ denoting an ensemble average carried out using the effective potential $V_{eff}(X[D])$.

In particular, in the discrete path integral method, each quantized nucleus is represented by a ring of *P* quasi-particles called beads. The coordinates for the *M*-quantized particles of the L-isotope are collectively denoted as **r**^L^ = {**r**_i_^L,q^; *i* = 1, …, *P*; *q* = 1, …, *M*}. Each bead associated with a given quantized particle is connected to its two neighbors via harmonic springs and is subjected to a fraction of the full classical potential. If we define the centroid positions for the *M* quantized particles $\left\{{\bar{r}}^{L}\right\}$ to be used as the principle variable, the quantum mechanical partition function of this hybrid quantized-classical system is given by [37]

(10) $$Q(X[L])=\int dS{\int d\bar{r}}^{L}{\left(\frac{P}{2\pi {\left(\lambda_{q}^{L}\right)}^{2}}\right)}^{3P/2}\int dRe^{-\beta V_{eff}(r^{L},S)}$$

where $\int dR=\int dr_{1}^{L}\cdots \int dr_{P}^{L}\delta ({\bar{r}}^{L})$, $V_{eff}(r^{L},S)$ is the effective quantum mechanical potential, **S** represents all classical coordinates of the solvent, the centroid coordinates are defined as ${\bar{r}}^{L,q}=1/P\sum_{i=1}^{P}r_{i}^{L,q}$, and the de Broglie thermal wavelength is $\lambda_{q}^{L}={\left(\beta \hbar^{2}/m_{q}^{L}\right)}^{1/2}$ for a particle of mass $m_{q}^{L}$.

In PI-FEP, [34] we further separate the path-integral average in Equation (9) into a hybrid classical molecular dynamics and quantum mechanical path-integral simulation [35,37]. The formalism is exact, [34,38,39,40] which was originally described by Feynman. In practice, the separation of phase-space sampling and path integral calculation of NQE into two distinctive steps facilitates the overall convergences, making it feasible to sample a large amount of solvent configurations of a condensed-phase system via classical MD, which would be more challenging using path-integral simulation directly [6]. Specifically, the ratio of the distribution functions due to an isotope substitution is determined using [34]

(11) $$\frac{Q(X[H])}{Q(X[D])}=\frac{<<e^{-\beta \{\Delta \bar{U}(r^{H},S)-\Delta \bar{U}(r^{D},S)\}}e^{-\beta \Delta \bar{U}(r^{D},S)}{>}_{FP,X[D]}{>}_{U(\bar{r},S)}}{<<e^{-\beta \Delta \bar{U}(r^{D},S)}{>}_{FP,X[D]}{>}_{U(\bar{r},S)}}$$

where the subscripts *X*[D] specifies that the ensemble averages are done using the ^2^H isotope, ∆*U*(**r**^D^, **S**) is the difference between the average potential of the *P*-discrete beads of the heavy isotope and that of the centroid coordinates, which is identical for both isotopes in the classical world, and $\Delta \bar{U}(r^{H},S)$ is the average difference potential of the light (hydrogen) atom.

(12) $$\Delta \bar{U}(r^{L},S)=\frac{1}{P}\sum_{q}^{M}\sum_{i}^{P}\left\{U(r_{i}^{L,q},S)-U({\bar{r}}^{q},S)\right\}$$

The average over free particle (FP) sampling is given by [37]

(13) $$<\cdots {>}_{{}_{FP,X[L]}}=\frac{\int dr_{P}^{L}\;\{\cdots \}\;\delta (\bar{r})e^{-[P/2{\left(\lambda_{m}^{L}\right)}^{2}]\sum_{i}^{P}{\left(\Delta r_{i}^{L}\right)}^{2}}}{\int dr_{P}^{L}\;\delta (\bar{r})e^{-[P/2{\left(\lambda_{m}^{L}\right)}^{2}]\sum_{i}^{P}{\left(\Delta r_{i}^{L}\right)}^{2}}}$$

where $\Delta r_{i}^{L}=r_{i}^{L}-r_{i+1}^{L}$.

Since free-particle distribution is known exactly at a given temperature, each ring-polymer distribution is generated according to that distribution and thus 100% accepted. Furthermore, in this construction, each new configuration in path integral sampling is created independently from the previous distribution, starting from a single initial bead position, allowing the ring-polymer configurations to move into completely different regions of configurational space (unlike Metropolis sampling or conventional molecular dynamics) [34]. This latter point is especially important to achieve rapid convergence by avoiding being trapped in a local minimum of the classical potential in path integral sampling [20,41,42,43,44,45]. This method described here has been implemented in the Q6 software [46].

In addition, we used a modified bisection sampling scheme for an enclosed ring-polymer, [40,41] which was originally designed for free-particle sampling of an open path [47,48]. Then, the relative positions of the perturbed light (H) isotope positions are related to the heavier (D) ones to be used in FEP-US calculations (Equation (11)) by

(14) $$\frac{r_{i}^{D,q}}{r_{i}^{H,q}}={\left(\frac{m_{q}^{H}}{m_{q}^{D}}\right)}^{1/2};\;i=1,\;2,\;\cdots ,\;P;q=1,\;2,\;\cdots ,\;M$$

where $r_{i}^{H,q}$ and $r_{i}^{D,q}$ are the coordinates for bead *i* of the corresponding H and D isotopes of the classical atom *q*, and $m_{q}^{H}$ and $m_{q}^{D}$ are the masses for the light and heavy nuclei [34].

## 3. Computational Details

We first performed Newtonian molecular dynamics simulations of the aqueous solution of one solute in a box of about 54 × 54 × 54 Å^3^, consisting of 4900–5000 water (H_2_O) molecules, for each of the acids included in this work. A total of 21 organic acids have been considered, focusing on oxygen titratable groups. A combined quantum mechanical and molecular mechanical (QM/MM) potential was employed, in which the solute molecule is modeled by the semiempirical Austin model 1 (AM1) method [49], and the solvent is represented by the three-point charge TIP3P model for water [50]. Since we are interested in the relative NQE of different compounds rather than the precise accuracy of the electronic structure, the use of a semiempirical potential is adequate in the present study. All simulations are performed using periodic boundary conditions along with the isothermal isobaric (NPT) ensemble at 298K and 1 atm using CHARMM [51]. Long-range electrostatic interactions are determined by using a particle-mesh Ewald method designed for combined QM/MM simulations, [52] whereas van der Waals terms are smoothed to zero at the same real space distance (15 Å) in the PME calculations. The geometry of water is constrained to the experimental value using the SHAKE algorithm, [53] whereas no geometrical constraints were applied to the solute molecules. We used the leap-frog integration scheme at a time step of 1 fs [51]. For each case, an initial equilibration was performed for 200 picoseconds, which is sufficient for the present simple solution cases, followed by 2 nanoseconds averaging, recording the coordinates in every picosecond for path integral simulations.

To determine nuclear quantum effects in acid–base equilibrium, we performed path integral (PI) simulations over the configurations saved in the classical molecular dynamics trajectory, employing free-energy perturbation theory (FEP) by treating the expansion of the de Broglie wavelength as a perturbation variable from the deuterium-isomer to that of the hydrogen atom [34]. In the double averaging simulation approach (Equation (11)), the relative free energy difference between the quantized solute and classical mechanical particles is determined by free-particle sampling in the inner average (Equation (11)). The ratio of the partition functions, i.e., deuterium isotope effects, is obtained via FEP, which is weighted by reference state (denominator of Equation (11)). To ensure fast convergence, we used the bisection sampling scheme with mass perturbation by enforcing the centroid coordinates of each quantized atom to the values sampled in the classical molecular dynamics simulation (outer average of Equation (11)) [41].The differential quantum mechanical spread between hydrogen and deuterium is related by their de Broglie wavelengths (or mass perturbation) in Equation (14), and the same spatial vectors $\theta_{i}^{q}$ for the two isotopes are used in the perturbation calculation. Previously, we have analyzed the dependence of numerical convergence with respect to the number of beads used in the ring-polymer representation for each quantized particle, and we found that *P*= 32 is a reasonable choice that balances precision and computational efficiency, and it is used in the condensed-phase calculations [20,54]. Of the 2000 configurations saved from the classical trajectory, we quantized the acidic hydrogen (deuterium) atom and other atoms that are within two covalent bonds and performed 1000 steps of free particle sampling for a total of 2,000,000 configurations.

Bimolecular complexes of each acid as a donor and its conjugate base as a hydrogen-bond acceptor with a water molecule have been optimized using density functional theory with the M06-2X functional and aug-cc-pVTZ and aug-cc-pVDZ, the latter for large molecules. Hydrogen bonding energies are determined at the M06-2X/aug-cc-pVTZ level of theory [55]. For each complex, we have carried out PI-FEP simulations to determine the effects of isotope substitution associated with the solvent molecule. In particular, to estimate the medium effect for each acid–base equilibrium, three separate simulations are performed, corresponding to isotope displacements (isotope effects) in the following pattern: (a) $AH(D)\cdots {\mathrm{OH}}_{2}$, (b) $AH[D]\cdots {\mathrm{OH}[D]}_{2}$, and (c) $A^{-}\cdots H[D]\mathrm{OH}[D]$, where the symbol D indicates that the hydrogen atom in front of the square brackets will be replaced by the deuterium isotope to yield the ratio of their partition functions (Equation (11)). The simulation for each of these configurations consists of 100,000 configurations of path integral sampling. Isotope substitution (a) is used as a reference with respect to the condensed phase simulations for the exchange effect, and patterns (b) and (c) are used to assess solvent molecule isotope effects as well as hydrogen-bonding delocalization. In all, the medium effects are determined according to Equation (7a). All path integral simulations are carried out using CHARMM in which the PI-FEP method has been implemented [34,51].

## 4. Results and Discussion

Table 1 contains the computed free-energy differences (Equation (6a)) between the acid DA and HA in water to account for the exchange effect (EE) and the free-energy contributions (Equation (7a)) due to media effects (ME), along with the computed and experimental isotope effects on acidity constants $\Delta pK_{a}$. Since there is no precise way of computing the free energy difference for the isotope substitution of a proton in water by a deuteron in D_2_O, we have used acetic acid as the reference compound to yield the reference free energy ∆*G*(LB) = ∆*G*(AcOL) = –0. 8975 kcal/mol, for determining the acidity shifts of all other acids in Table 1. This is accomplished by subtracting the computed exchange isotope effect in AcOH → AcOD in water from that of the experimental $\Delta pK_{a}$ according to Equation (4) (Table 1) [4,17,19,25]. Implicitly, the small solvent effects on the EE term and the ME effects are also lumped into this reference value as a constant.

**Figure 2:**
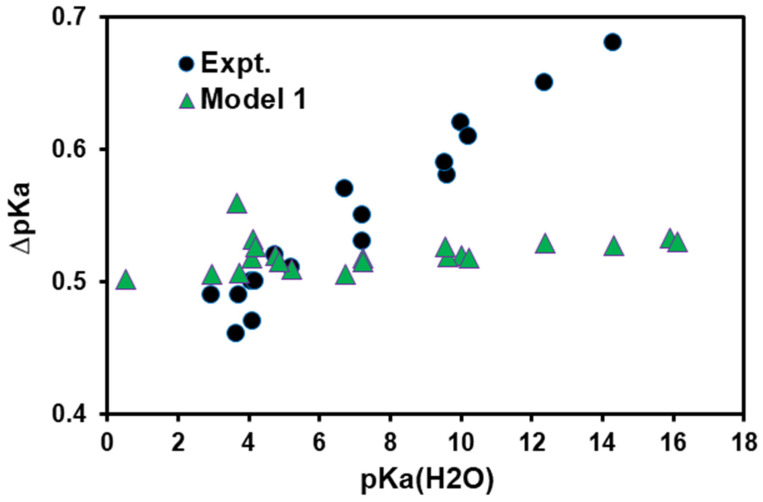
Experimental and computed isotope effects on acidity using Model 1 and path integral-free energy perturbation simulations.

Figure 2 and the corresponding data in Table 1 show that the computed Model 1 isotope effects on acidity of a range of 21 acids containing alcohol, phenol, and carboxyl functional groups are nearly constant with an average value of 0.52 ± 0.01 pKa units. This is in reasonable accord in comparison with the average experimental value, 0.55 ± 0.06 (Table 1). Nevertheless, Figure 1 shows that the trend of increasing $\Delta pK_{a}$ with $pK_{a}^{H_{2}O}$ is absent in the computed values employing the Model 1 method (Equation (6a)), which considers only the isotope exchange effect, although the average $\Delta pK_{a}$ shift is in agreement with experiments. This behavior is analogous to that of the computational results from density functional theory coupled with the polarizable continuum solvation model (PCM) displayed in Figure 1 from the data in ref. [19]. Effectively, that computational approach mirrors the present Model 1. The findings from the present study and that of ref. [19] show that the dominant effect, the exchange of a proton at the acidic site by deuteron, is captured by the change in zero-point energies as a result of vibrational frequency shift, but additional factors dictate the trends.

Mora-Diez et al. carried out a thorough analysis of various factors that affect the computed $\Delta pK_{a}$ [19]. First, employing a solvent density-corrected continuum model for D_2_O, the authors showed that the average energy difference $\Delta E_{sol}^{D_{2}O}(\mathrm{DA})\;-$ $\Delta E_{sol}^{H_{2}O}(\mathrm{HA})$ and the relative free energy of formation of the conjugate bases $\Delta G_{f}^{D_{2}O}(A^{-})-\Delta G_{f}^{H_{2}O}(A^{-})$ are respectively 0.000 and −0.006 kcal/mol, which is consistent with the postulate of negligible contributions from these terms by Schowen and Schowen [1].This approximation is adopted in Model 1 in this work. Mora-Diez et al. further analyzed entropic and thermal energy contributions to the total Gibbs energy difference of the isotope effects, and they concluded that they also nearly cancel out at ambient temperatures, reaching the conclusion that accurate predictions can be made from zero-point-energy differences between DA and HA in the respective solvents [19]. These analyses highlight the reason for successfully obtaining a reasonable agreement on average with experiment on the basis of Model 1 methods.

**Figure 3:**
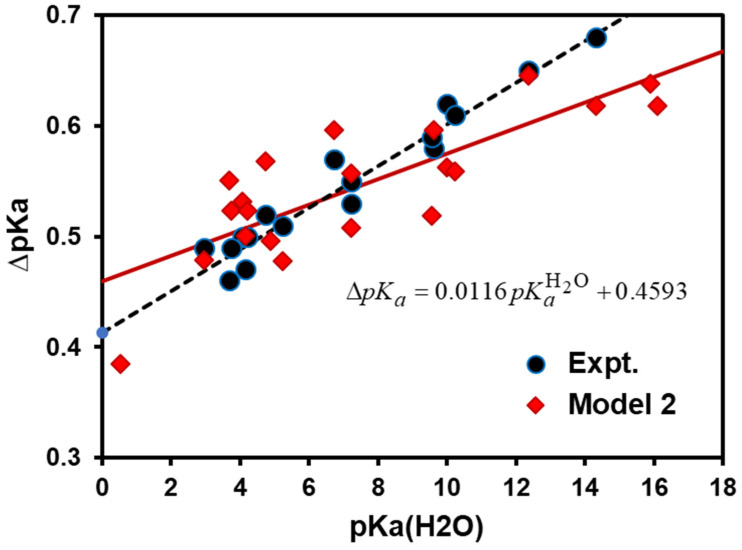
Experimental and computed isotope effects on acidity using Model 2 and path integral-free energy perturbation simulations. Medium isotope effects on specific acid–base interactions with the solvent are included in Model 2.

The computed ∆*pK_a_* using Model 2 (Equation (7a)), which includes the medium effect on acid–base equilibrium, is plotted in Figure 3. In comparison with Figure 2, the trends of ∆*pK_a_* with increasing acidity is clearly captured by Model 2. The average value of the ∆*pK_a_* shifts included in this work is 0.55 ± 0.06, which is in agreement with the experimental data (0.55 ± 0.06). Linear regression of the computed data yields a relationship of ∆*pK_a_* = 0.0116*pK_a_*^H_2_O^ + 0.4593, which may be compared with the experimental trend based on the data in Table 1: ∆*pK_a_* = 0.0188*pK_a_*^H_2_O^ + 0.4127. Interestingly, such linear free-energy relationships have frequently been discussed, including the extensive work of Bell and Kuhn, [3] who obtained a slope of 0.017 and an intercept of 0.44 for oxygen-containing organic acids, and that by Salomaa et al. for inorganic oxyacids (slope = 0.034, and intercept = 0.30) [16]. The slope from the experimental data is steeper than that from the theoretical model. One possible source of errors in the present computational study is to have only included one solvent molecule in path integral simulations. Nevertheless, it is significant that the present study unequivocally showed that the trend of $pK_{a}^{H_{2}O}$ dependence of the solvent isotope effects on the acid–base equilibrium constant is due to the medium effect, originating from the isotope effects in the specific hydrogen bonding interactions of the acid and its conjugated base to donate and to accept a hydrogen bond from the solvent H_2_O or D_2_O.

**Figure 4:**
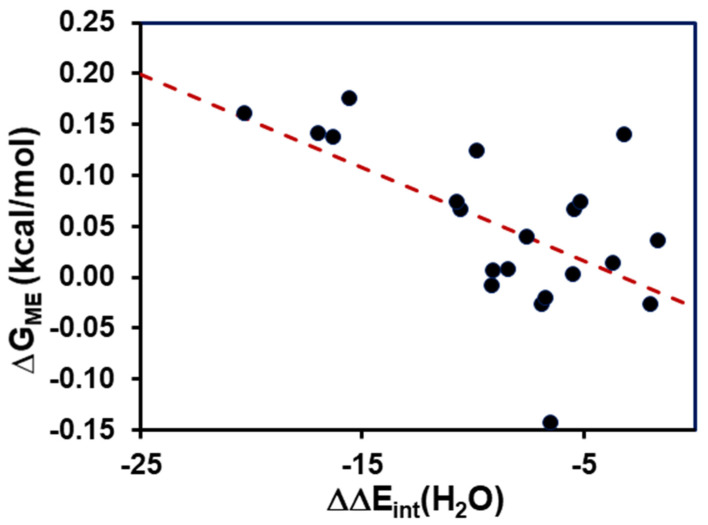
Correlation of the total isotope medium effects (∆G_ME_) on acid and base equilibrium with the difference in hydrogen-bonding energies between the conjugate base and acid, ∆∆E_int_ = ∆E_HB_(A^−^…HOH)—∆E_HB_(AH…OH_2_), due to the replacement of a water (H_2_O) by a heavy water (D_2_O) molecule. Energies are given in kcal/mol.

To further understand the origin of the medium effects, we have determined the hydrogen-bonding interaction energies for the donor and acceptor hydrogen bonds of the acid $\mathrm{AH}\cdots {\mathrm{OH}}_{2}$ and its conjugated base $A^{-}\cdots \mathrm{HOH}$, respectively (Table 2). Here, the M06-2X/aug-cc-pVTZ energies are used for analysis. Although the $pK_{a}^{H_{2}O}$ dependence of the computed isotope effects on $pK_{a}$ change is clearly revealed in Figure 3, we found that it is the hydrogen-bonding energy of the conjugated base with the solvent that shows the dominant correlation with the total free energy changes due to medium effects (Table 2). Hydrogen-bonding donating effects on a water molecule play a minor role without apparent trends (Table 2). In Figure 4, we illustrate the dependence of the computed total medium effects in terms of free energy with the combined hydrogen-bonding effects by taking the difference of the computed binding energies between the conjugated base (A^−^) and the acid (AH) with a water molecule: $\Delta \Delta E_{\mathrm{int}}=\Delta E_{HB}(A^{-}\cdots \mathrm{HOH})-$$\Delta E_{HB}(\mathrm{AH}\cdots {\mathrm{OH}}_{2})$. The correlation between $\Delta G_{\mathrm{ME}}$ and $\Delta \Delta E_{\mathrm{int}}$ is clear, though not perfect, including several apparent outliers. We did not further inquire into the origin of the deviations, but the trend in Figure 4 is sufficient to illustrate the key factors through a linear free-energy relationship responsible for the sloped $pK_{a}$ isotope effects on the acidity strength. Therefore, we conclude that the specific intermolecular interactions of the solvent molecule donating hydrogen bonds to the conjugated base plays the key role of determining the observed increase in $pK_{a}$ shifts with increasing $pK_{a}^{H_{2}O}$ as the solvent changes from water to heavy water.

## 5. Concluding Remarks

Deuterium isotope effects on the acid–base equilibrium of a series of organic acids consisting of alcohols, phenol derivatives, and carboxylic acids have been determined using a mixed path integral and free-energy perturbation simulation method. Following the classification of the intrinsic isotope exchange effect of the acid itself and the medium effect due to solute–solvent interactions by Schowen and Schowen, two theoretical models are described in the present study. In Model 1, we only consider the exchange effect by computing the ratio of the partition functions of the acid with a proton or a deuteron at the titratable site in water. This follows the hypothesis that the medium effect is “vastly less important” than the exchange effect (mostly due to difference in zero-point energy) [1]. A recent comprehensive study by Mora-Diez et al. also follows the Model 1 framework, but density functional theory calculations with optimized structures in continuum media were used in that work. Both the present path integral-free energy perturbation simulation and the previous calculations with density functional theory yield an apparent agreement with experimental data in terms of average $pK_{a}$ shifts. However, the linear dependence of ∆*pK_a_* vs. acidity constant observed experimentally was not captured. In Model 2, explicit solute–solvent hydrogen bonding interactions are considered with isotope substitutions also in the solvent molecule to estimate the medium effect in path integral simulations. We found that the linear free-energy relationship between $\Delta pK_{a}$ and $pK_{a}^{H_{2}O}$ can be reproduced by including contributions from the medium isotope effect. Therefore, even though the dominant factor leading to characteristic deuterium isotope effects on the acidity of specific functional groups is due to the difference in zero-point energy as a result of isotope exchange of the acid itself, the small variations with $pK_{a}^{H_{2}O}$ values originate from detailed hydrogen bonding interactions with the solvent. We further analyzed the relative contributions due to the hydrogen-bond donation from the acid and hydrogen-bond acceptor by the conjugate base, and we found that the former does not exhibit a clear trend, whereas anion–water hydrogen-bonding energies have stronger correlation with the ∆*pK_a_* slope than acid–water complexes. The present work illustrates the significance of considering all factors of the intrinsic exchange effect of the acid and the medium contributions to obtain both average deuterium isotopic effects on $pK_{a}$ and its dependence on the acidity in water.

## Figures and Tables

**Table 1:** Computed free energy differences (kcal/mol) of isotope exchange effects (EE) and medium effects (ME), and $pK_{a}$ differences of organic acids in D_2_O and H_2_O along with experimental data and $pK_{a}$ values in water at 25 °C.

	Expt.		Calc. ^b^	PI-FEP		$\Delta pK_{a}(\mathrm{Calc}.)$	
Acid (HA)	$pK_{a}^{\mathrm{HA}}$	$\Delta pK_{a}$	$\Delta pK_{a}$	$-\Delta \Delta G_{\mathrm{EE}}^{w\to d}$	$\Delta \Delta G_{\mathrm{ME}}^{w\to d}$	Model 1	Model 2
MeCO_2_H	4.75 ^a^	0.52 ^a,b^	0.62	1.4988	0.0656	0.52	0.57
HCO_2_H	3.67 ^c^	0.46 ^c^		1.5360	0.0052	0.55	0.55
EtCO_2_H	4.88 ^d^			1.4754	−0.0094	0.50	0.50
F_3_CCO_2_H	0.52 ^e^			1.4583	−0.1444	0.49	0.38
PhCO_2_H	4.21 ^f^	0.50 ^f^		1.4909	0.0127	0.51	0.52
β-NaphCO_2_H	4.14 ^g^	0.47^g^	0.61	1.4994	−0.0274	0.52	0.50
EtOH	15.9 ^h^			1.4998	0.1605	0.52	0.64
PrOH	16.1 ^i^			1.4966	0.1362	0.52	0.62
2-ClEtOH	14.3 ^j^	0.68 ^j^		1.4925	0.1401	0.52	0.62
CF_3_CH_2_OH	12.4 ^k^	0.65 ^k^		1.4953	0.1742	0.52	0.65
PhOH	10.0 ^g^	0.62 ^g^	0.63	1.4913	0.0656	0.51	0.56
3-MeOPhOH	9.62 ^g^	0.58 ^g^	0.63	1.4805	0.1228	0.51	0.60
4-MeOPhOH	10.2 ^g^	0.61 ^g^	0.62	1.4793	0.0726	0.51	0.56
2-NitroPhOH	7.22 ^b,l^	0.55 ^b,l^	0.68	1.4801	0.0018	0.51	0.51
4-NitroPhOH	7.22 ^g^	0.53 ^g^	0.63	1.4757	0.0733	0.50	0.56
3,5-diNitroPhOH	6.73 ^m^	0.57 ^a,b^	0.62	1.4629	0.1394	0.49	0.60
2,5-diNitroPhOH	5.22 ^g,n^	0.51 ^g,n^	0.69	1.4681	−0.0271	0.50	0.48
2,4-diNitroPhOH	4.07 ^g,n^	0.50 ^b,g^	0.68	1.4794	0.0355	0.51	0.53
2,6-diNitroPhOH	3.73 ^n^	0.49 ^n^	0.67	1.4647	0.0392	0.50	0.52
4-Cl-2,6-diNitroPhOH	2.96 ^a,n^	0.49 ^a,n^	0.67	1.4631	−0.0209	0.49	0.48
β-Naphthol	9.55 ^g^	0.59 ^g^	0.64	1.4907	0.0065	0.51	0.52

a. Refs. [4,25]. b. Ref. [19]. c. Ref. [17]. d. Ref. [26]. e. Ref. [27]. f. Refs. [1,14]. g. Ref. [15]. h. Ref. [28]. i. Ref. [29]. j. Ref. [30]. k. Ref. [31]. l. Ref. [32]. m. Ref. [33]. n. Ref. [3].

**Table 2:** Computed interaction energies (kcal/mol) for the acid (AH) donating a hydrogen bond to a water molecule and for the conjugated base $A^{-}$ accepting a hydrogen bond. Hydrogen bonding energies are determined using M06-2X/aug-cc-pVTZ.

Acid (HA)	$\Delta E_{\mathrm{HB}}{(\mathrm{AH}}^{}\cdots {\mathrm{OH}}_{2})$	$\Delta E_{\mathrm{HB}}{(A}^{-}\cdots \mathrm{HOH})$	$\Delta \Delta G_{\mathrm{ME}}^{w\to d}$
MeCO_2_H	−11.1	−16.5	0.0656
HCO_2_H	−11.2	−20.2	0.0052
EtCO_2_H	−11.1	−20.2	−0.0094
F_3_CCO_2_H	−10.5	−17.0	−0.1444
PhCO_2_H	−11.3	−14.9	0.0127
β-NaphCO_2_H	−11.3	−18.1	−0.0274
EtOH	−5.0	−25.2	0.1605
PrOH	−3.5	−19.8	0.1362
2-ClEtOH	−5.9	−22.9	0.1401
CF_3_CH_2_OH	−7.0	−22.5	0.1742
PhOH	−7.0	−17.5	0.0656
3-MeOPhOH	−6.9	−16.7	0.1228
4-MeOPhOH	−6.8	−17.5	0.0726
2-NitroPhOH	−8.8	−14.2	0.0018
4-NitroPhOH	−8.7	−13.8	0.0733
3,5-diNitroPhOH	−9.9	−13.0	0.1394
2,5-diNitroPhOH	−10.2	−12.2	−0.0271
2,4-diNitroPhOH	−10.3	−12.0	0.0355
2,6-diNitroPhOH	−5.6	−13.2	0.0392
4-Cl-2,6-diNitroPhOH	−5.9	−12.6	−0.0209
β-Naphthol	−7.2	−15.6	0.0065

## Data Availability

All input and derivative data are available up on request from the authors.

## Sample Availability

Computational input samples are available from the authors.
